# Prevalence and Risk Factors of Upper Extremity Injuries in Indoor Bouldering: A Cross-Sectional Study

**DOI:** 10.7759/cureus.69729

**Published:** 2024-09-19

**Authors:** Lucas Mena, Leonardo Zanesco, Jorge H Assunção, Caio Checchia, Mauro Gracitelli, Eduardo A Malavolta

**Affiliations:** 1 Orthopaedics and Traumatology, Hospital das Clínicas da Faculdade de Medicina da Universidade de São Paulo (HCFMUSP), São Paulo, BRA; 2 Orthopaedics, Diagnósticos da América (Dasa) Hospital Nove de Julho, São Paulo, BRA; 3 Orthopaedics, Hospital Sírio-Libanês, São Paulo, BRA; 4 Orthopaedics, Hospital do Coração (Hcor), São Paulo, BRA

**Keywords:** athletic injuries, climbing, orthopaedic surgery shoulder and elbow and upper extremity, orthopedic sports medicine, shoulder joint pain, sport injury, sports problems

## Abstract

Objectives: To describe the prevalence and risk factors associated with upper extremity injuries among indoor bouldering practitioners, focusing on dynamic movements and specific training methods.

Methods: We conducted a cross-sectional study with 35 indoor bouldering climbers from a metropolitan area. Data were collected through an adapted online questionnaire and in-person orthopedic evaluations by certified specialists. Statistical analysis was performed using Stata 18 (StataCorp LLC, College Station, TX), calculating the prevalence of pain and injuries and associations between dynamic movements and specific injuries.

Results: The sample was predominantly male (80%, n = 28/35), with a mean age of 25.9 years. Shoulder anterior apprehension was significantly associated with dynamic climbing styles (p = 0.028), with a prevalence difference of 0.3 (95% CI: 0.04, 0.57). Finger pulley and shoulder injuries affected 22.9% (n = 8/35) and 25.7% (n = 9/35) of participants, respectively.

Conclusions: Our study found a significant association between dynamic movements and upper extremity injuries in bouldering climbers, highlighting the pressing need for injury prevention strategies. Despite climbing being an overhead sport, our findings suggest distinct pathophysiology from the thrower’s shoulder, necessitating further investigation.

## Introduction

Bouldering, a unique discipline of rock climbing, involves short, challenging routes without ropes or harnesses. It is distinct from other climbing activities, as it is practiced indoors on artificial climbing walls and outdoors on natural boulders, and it requires strength, agility, and strategic problem-solving. Bouldering's dynamic and intense nature exposes climbers to specific physical demands, particularly on the upper extremities. With the sport's growing popularity, especially after its inclusion in the Olympic Games, understanding the prevalence and risk factors of related injuries is crucial for orthopedic practitioners.

Recent studies have highlighted the commonality of upper extremity injuries among climbers. Auer et al. (2021) [1] and Müller et al. (2022) [2] documented high prevalence rates of shoulder and finger injuries, the last being based on data from emergency departments, which may not capture the full spectrum of chronic injuries and subclinical conditions. Other studies highlight chronic pathologies such as tendinopathy and overuse injuries resulting from repetitive stress [3,4]. Despite these findings, limited research explicitly addresses how climbing techniques and training regimens influence injury prevalence and prevention in indoor bouldering [5].

While shoulder injuries in overhead sports like baseball often involve glenohumeral internal rotation deficit (GIRD) [6-8], the biomechanics of bouldering differ significantly. In contrast, bouldering requires various movements of multiple muscle groups. We hypothesized that climbers would not have the same shoulder adaptations found in overhead throwers.

This study aims to conduct a cross-sectional analysis of injuries in indoor bouldering practitioners, focusing on the prevalence and risk factors associated with dynamic climbing movements. It also seeks to provide insights into injury prevention and rehabilitation strategies.

## Materials and methods

Study design

This cross-sectional study investigates upper extremity injuries among indoor boulder practitioners. An initial online interview with participants gathered information on demographics, climbing experience, and injury history. Then, an in-person orthopedic evaluation was conducted. The study population consisted of indoor boulder climbers from a metropolitan region. Data were collected from September 2023 to May 2024. The internal board ethics committee approved the study, and all participants provided informed consent to participate.

Participants

Participants were recruited through a non-probabilistic convenience sampling method, primarily from climbing gyms. This approach ensured easy access to a relevant population while aiming to capture a broad spectrum of indoor boulder climbers. Snowball sampling was also employed. Participants were encouraged to appoint other eligible climbers, allowing for a more varied participant pool. The study focused on rock climbers who predominantly used indoor bouldering as their primary modality. It included both recreational and professional climbers with diverse experience and skill levels.

Both male and female climbers between the ages of 15 and 65 years were eligible to participate, having at least six months of bouldering experience. Regularity in climbing practice was also essential; participants were required to climb at least twice a week or accumulate a minimum of 10 hours of climbing per month. Exclusion criteria included participants with irregular climbing frequency.

Data sources

We used an adapted version of the questionnaire developed by Auer et al. (2021) [1], initially designed to assess climbing-related activities and injuries. The questionnaire was translated from German to Portuguese to ensure accessibility and relevance to the Brazilian climbing community. Before the study, we consulted with local climbers to confirm its understandability and cultural relevance. Our questionnaire is publicly available [9].

Demographic and lifestyle measurements were also critical components of the assessment. Anthropometric data, such as height, weight, and BMI, were self-recorded. Participants provided information regarding lifestyle factors, such as the use of alcohol, drugs, and tobacco.

**Figure 1:**
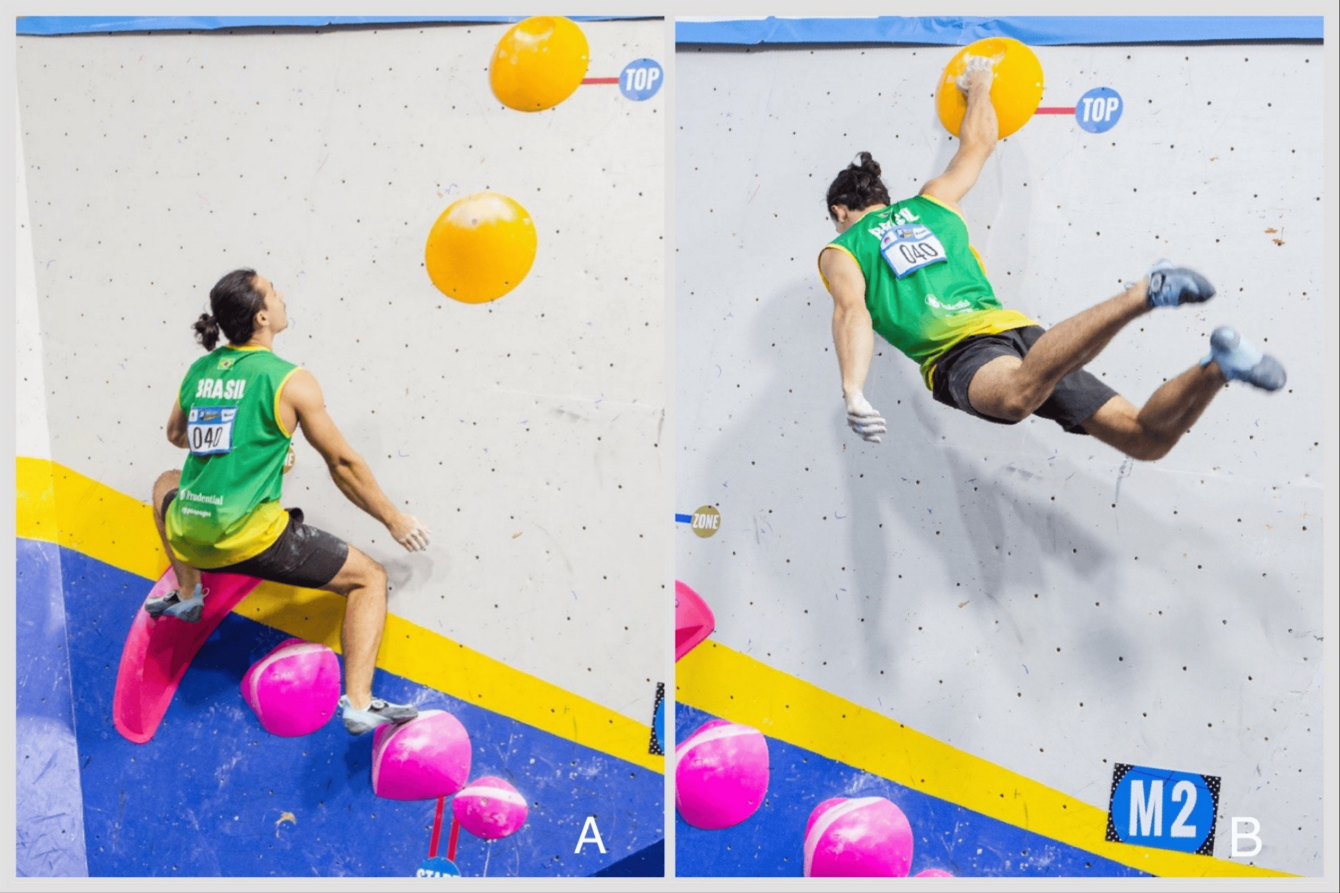
Preparation and execution of a dynamic movement. Figure 1 is from the personal collection of photographer Aline Machado, and the rights were granted for use in this publication.

Climbing experience was measured by total years and weekly session frequency. The climbing difficulty was assessed using V-level [10]. We also explored preferences for dynamic (Figure 1) versus static movements to determine their impact on injury risk. Training practices, such as app-controlled routes like Kilter Board (Setter Closet, Boulder, CO) and Moon Board (Moon Climbing, Sheffield, UK), and equipment (fingerboards [11] and campus boards [11]) were evaluated.

After completing the online questionnaire, participants attended an in-person evaluation by last-year orthopedic surgery residents. This evaluation served to cross-check and expand upon the data collected via the questionnaire, beginning with thoroughly verifying the participant's medical history.

Specialists performed a comprehensive physical examination focused on the shoulder, elbow, wrist, and hand, assessing the range of motion (ROM) and detecting abnormalities. The physical examination focused on the upper extremities following standard practice. We conducted static and dynamic inspections, palpation, ROM tests, and specific assessments following standard practice [12,13]. Patients who reported experiencing apprehension events accompanied by pain and climbing performance limitation lasting two weeks or more were diagnosed with glenohumeral instability when confirmed by physical examination.

The ROM of the shoulder, elbow, and wrist was measured with a goniometer. For shoulder internal and external rotation, we evaluated the ROM with the shoulder abducted at 90° with scapular stabilization [12].

The study's primary outcomes focused on confirmed diagnosis, pain location, and intensity, with participants reporting both the areas and severity of pain experienced. The severity was assessed based on its impact on their ability to climb, ranging from no pain to some pain while climbing, to an inability to climb due to the intensity of the pain.

Statistical analysis

Data Preparation

Data were processed using Stata 18 BE (StataCorp LLC, College Station, TX), with raw measurements transformed into categorical variables when necessary, such as climbing frequency, difficulty levels (V-level), and demographic factors. Continuous variables like age, years of climbing, and BMI were used directly in some analyses.

We used prevalence and difference ratios to evaluate the associations between climbing-related factors and specific injury outcomes. This method estimated the likelihood of conditions within the study population, providing a measure of how common an injury is among those exposed to certain risk factors compared to those who are not.

Statistical Software and Techniques

Descriptive statistics provided an initial overview of the data, while unadjusted analyses, such as t-tests and chi-square tests, were used to compare baseline characteristics between groups. Fisher's exact test was mainly utilized for categorical data with small sample sizes to ensure accurate p-values. The E-value [14] evaluates the robustness of associations against potential unmeasured confounding.

Analysis of Shoulder Subluxation and Climbing Style

Given our small sample size, the relationship between the positive anterior shoulder apprehension test during climbing and dynamic climbing movements was analyzed using Fisher's exact test. We used E-value to evaluate the robustness of the observed association between dynamic climbing style and shoulder subluxation. The E-value [14] quantifies the minimum strength of association that an unmeasured confounder would need to have with both the exposure (dynamic movement) and the outcome (shoulder apprehension) to account for the observed association fully. This approach provides insights into the potential impact of unmeasured confounding on the study's findings.

Sample Size and Power Calculations

The study aimed to include a sample size of 35 participants, calculated to achieve 80% confidence with a 5% margin of error. This calculation was based on an expected population proportion of 6.3% for shoulder injuries among climbers [2].

Use of AI tools

AI language models, such as ChatGPT, Consensus, and Grammarly, were used to assist in writing and refining the English content of this manuscript. The output was entirely reviewed and edited by the authors.

## Results

A total of 35 indoor bouldering climbers, with a majority of 80% (n = 28) identifying as male, were included in this study. The mean age of the participants was 25.9 years (SD = 8.2) (range = 17-60). The average height was 1.73 meters (SD = 0.07), and the mean weight was 67 kg (SD = 9.7), leading to an average BMI of 22.2 (SD = 2.3) (Table 1).

**Table 1:** Demographic characteristics and substance use. Table 1 presents the demographic characteristics of the climbers, including age, gender, and substance use patterns such as alcohol and tobacco consumption.

Variable	Frequency (n)	Mean/proportion (%)	Std. Dev.	Min	Max
Age (years)	35	25.9	8.2	17	60
Biological sex	.	.	.	.	.
Male	28/35	80%	.	‘	.
Female	7/35	20%	.	.	.
Height (m)	35	1.7	.07	1.5	1.9
Weight (kg)	35	67.0	9.66	43	86
BMI (kg/m²)	35	22.2	2.34	17.2	26.5
Smoking (yes, %)	10/35	28,6%	.	.	.
Marijuana (yes, %)	13/35	37.1%	.	.	.
Alcohol (yes, %)	26/35	74.3%	.	.	.

Climber experience, weekly training hours, years of climbing, typical V-level on a regular training day, and the highest V-level achieved are presented (Table 2). Regarding training characteristics, 14 participants preferred dynamic climbing, with a prevalence of 40% (n = 14/35; 95% CI, 23.9% to 57.9%). Fingerboard training was more common, with 65.7% (n = 23/35; 95% CI, 47.8% to 80.8%) participants incorporating it into their routine. Similarly, campus board training was practiced by 60% (n = 21/35; 95% CI, 42.1% to 76.1%). The most prevalent training method was app-controlled routes, with 77.1% (n = 27/35; 95% CI, 59.9% to 89.6%) of participants utilizing this approach.

**Table 2:** Climbing characteristics. V-level [10].

Climbing level	Proportion/frequency	Hours per week	Years climbing	Baseline V-level	Maximum V-level
Beginner climber	5.7% (2/35)	4.5	1	V-2	V-4
Intermediate climber	57.1% (20/35)	5.8	4.7	V-4	V-6
Advanced climber	11.4% (4/35)	8.7	5.2	V-7	V-8
Elite climber	11.4% (9/35)	13.3	10.4	V-8	V-10

Range of motion

ROM measurements for the dominant and non-dominant limbs in climbers showed minimal differences, indicating balanced joint flexibility. The ROM values for the shoulder (Table 3), elbow (Table 4), and wrist joints (Table 5) were similar bilaterally.

**Table 3:** Comparison of dominant and non-dominant range of motion of the shoulder.

Shoulder	Dominant mean (°) (SD)	Non-dominant mean (°) (SD)	Difference (°) (SD)	P-value (Wilcoxon signed-rank)
Flexion	189 (2.42)	191 (2.23)	-1.83 (0.19)	0.34
Extension	65 (2.38)	65 (1.90)	0.05 (0.48)	0.39
External rotation	100 (2.84)	99 (2.19)	0.94 (0.65)	0.74
Internal rotation	78 (2.81)	81 (1.67)	-3.05 (1.14)	0.64

**Table 4:** Comparison of dominant and non-dominant range of motion of the elbow.

Elbow	Dominant mean (°) (SD)	Non-dominant mean (°) (SD)	Difference (°) (SD)	P-value (Wilcoxon signed-rank)
Flexion	149 (1.76)	149 (1.48)	0.05 (0.28)	0.97
Extension	-1 (1.14)	0 (0.99)	-1.00 (0.15)	0.59
Pronation	97 (0.67)	90 (0.77)	6.72 (-0.10)	<0.001
Supination	90 (0.50)	90 (0.57)	-0.09 (-0.07)	0.82

**Table 5:** Comparison of dominant and non-dominant range of motion of the wrist.

Wrist	Dominant mean (°) (SD)	Non-dominant mean (°) (SD)	Difference (°) (SD)	P-value (Wilcoxon signed-rank)
Flexion	104 (2.78)	94 (1.71)	9.05 (1.07)	<0.001
Extension	87 (1.45)	83 (2.21)	4.43 (-0.76)	0.09
Radial deviation	29 (1.37)	27 (1.24)	2.20 (0.13)	0.13
Ulnar deviation	36 (0.92)	38 (0.82)	-1.71 (0.10)	0.05

Current pain locations

Shoulder pain was reported by 31.4% (n = 11), making it one of the most common complaints, second only to hand pain at 37.1% (n = 13). Conversely, spine and ankle/foot pain were less prevalent, each reported by 5.7% (n = 2). These findings are detailed below in Table 6. Among those with shoulder pain (n = 11), during physical examination, rotator cuff alterations were identified in 27.3% (3/11). Acromioclavicular (AC) joint pain in participants with shoulder pain without rotator cuff alterations was present in 25% (2/8).

**Table 6:** Current pain locations.

Pain location	Frequency (n)	Prevalence (%)	95% CI, lower	95% CI, upper
Hand	13	37.1	21.47	55.08
Shoulder	11	31.4	16.85	49.29
Elbow	4	11.4	3.20	26.74
Spine	2	5.7	0.70	19.16
Ankle and feet	2	5.7	0.70	19.16
No pain	12	34.3	19.13	52.21

Prevalence of injuries

Upper Limb Injuries

Finger pulley lesions affected 22.9% (8/35) of climbers, and shoulder injuries were reported by 25.7% (9/35). Shoulder apprehension during climbing was noted in 17.1% (6/35) of participants. Lateral epicondylitis was reported in 14.3% (5/35), and wrist ligament injuries were noted in 8.6% (3/35).

Lower Limb Injuries

Sprained ankles were reported by 5.7% (2/35) of participants, and knee injuries affected 8.6% (3/35) of climbers.

Other Conditions

Additionally, 2.9% (1/35) of participants reported lumbar pain, 8.6% (3/35) experienced minor muscle sprains, and previous fractures were rare, with 5.7% (2/35) of participants. Of the participants, 37.2% (13/35) reported no specific diagnosis (Table 7).

**Table 7:** Cumulative prevalence of injuries.

	Frequency (n)	Cumulative prevalence	95% CI
No diagnosis	13	37.2	22.5-54.6
Finger pulley lesion	8	22.9	11.6-40.2
Shoulder injury	9	25.7	13.6-43.2
Lateral epicondylitis	5	14.3	5.9-30.8
Knee injury	3	8.6	2.7-24.2
Wrist ligament injury	3	8.6	2.7-24.2
Minor muscle sprain	3	8.6	2.7-24.3
Sprained ankle	2	5.7	1.4-21.0
Fracture	2	5.7	1.4-21.0
Lumbar pain	1	2.9	0.4-18.8

Shoulder instability

A total of six out of 35 participants (17.1%) reported at least one episode of apprehension sensation, requiring a minimum of two weeks to recover before resuming climbing. All were male, with a mean age of 22 years and a mean height of 1.78 m. Notably, no cases of anterior luxation were reported in this group. Climbers with shoulder apprehension had an average of 4.5 years of climbing experience versus 6.5 years for those without (p = 0.021), indicating a significant relationship between less experience and injury risk.

**Figure 2:**
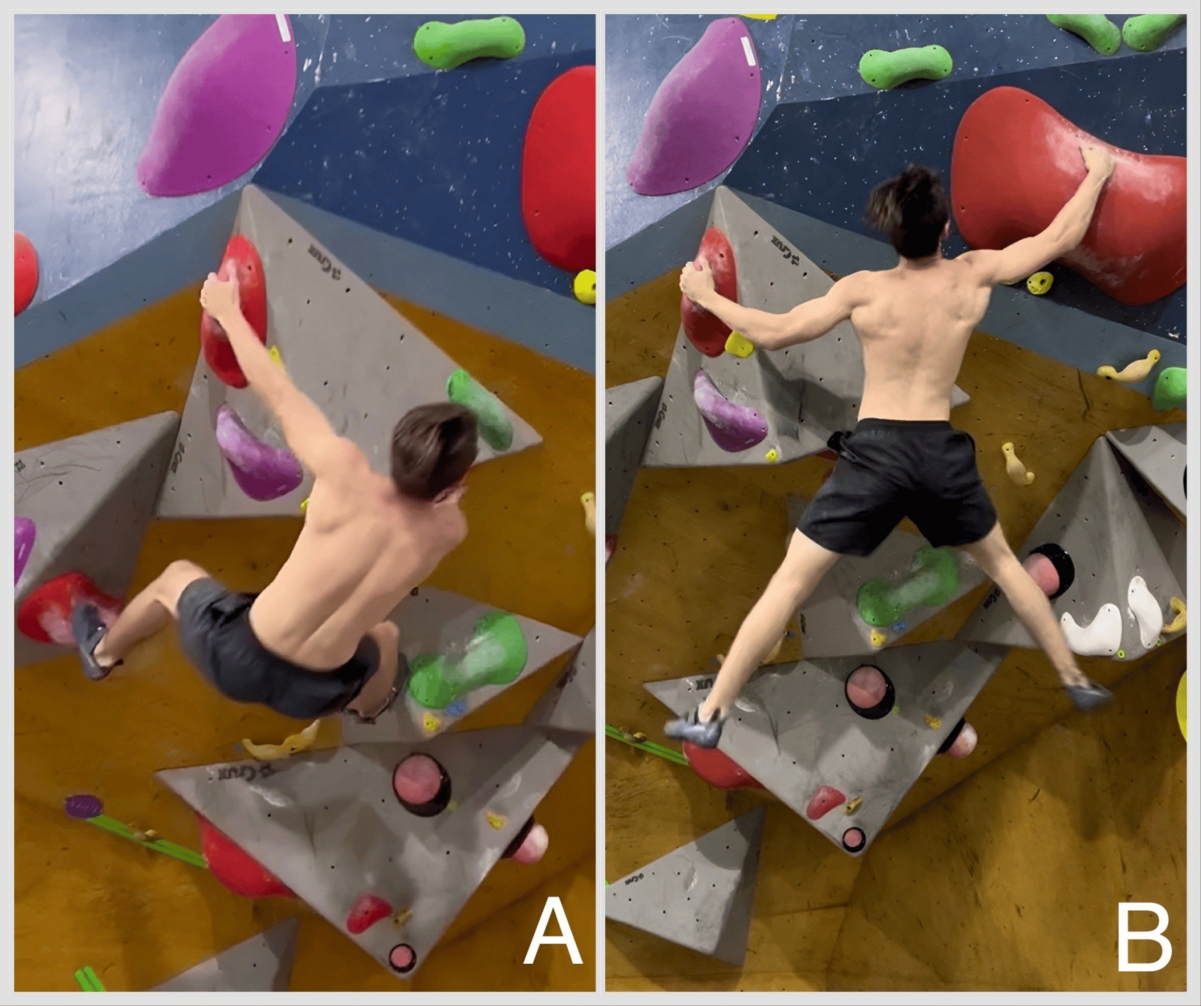
Climber with a history of previous shoulder apprehension performing a dynamic movement. Visible activation of shoulder muscles, including the latissimus dorsi and deltoids, demonstrates the complexity of shoulder engagement in climbing. This figure is a contribution from Lucas Avello, republished with permission.

A significant association was found with dynamic climbing movement preference (Figure 2), using Fisher's exact test (p = 0.028). This association was also present on the additive scale, with a cumulative prevalence difference of 0.3 (95% CI: 0.04, 0.57) and an e-value of 14.9. However, the multiplicative scale does not confirm it, as the cumulative prevalence ratio is 7.5 (95% CI: 0.98, 57.55).

The ROM for the dominant shoulder, including flexion, extension, internal rotation, and external rotation, was assessed in climbers with and without shoulder apprehension. No statistically significant differences were observed between the two groups across all measures, with all p-values indicating a lack of significance (Table 8).

**Table 8:** Comparative analysis of shoulder range of motion between climbers exhibiting and not exhibiting shoulder.

Range of motion	Mean (apprehension)	Mean (apprehension)	P-value (Wilcoxon rank-sum)
Flexion	185	190	0.34
Extension	70	72	>0.99
Internal rotation	69	79	0.20
External rotation	102	99	0.69

## Discussion

This cross-sectional study looks at upper limb injuries among indoor bouldering practitioners. The high prevalence of finger pulley injuries and shoulder injuries - each affecting 22.9% (n = 8/ 35) of participants - points to the unique physical demands of bouldering. Lateral epicondylitis was diagnosed in 14.3% (n = 5/35) of climbers, and wrist ligament injuries were reported in 8.6% (n = 3/35). Fractures, although rare (5.7%, n = 2/35), included a physeal fracture in an adolescent due to stress, and one ankle fracture from landing.

Hand injuries, particularly pulley injuries, emerged as a significant concern in our cohort, with 37.1% (n = 13/35) reporting hand pain and 22.9% (n = 8/35) diagnosed with pulley injuries. These figures are notably higher than those reported by Müller et al. (2022) [2] (0.7%) and Josephsen et al. (2007) [15] (9.8%). The discrepancy likely reflects the increased strain on finger tendons in bouldering, where repetitive gripping and dynamic moves can elevate the risk of tendon damage. Compared to other forms of climbing, bouldering may inherently place more extreme demands on the hands, leading to higher injury rates.

The 25.7% (n = 9/35) cumulative prevalence of diagnosed shoulder injuries, including anterior instability (17,1%, n = 6/35), is similar to the rates reported by Schöffl et al. (2015) [16] (17.2%) and Auer et al. (2021) [1] (16%). Shoulder pain was frequently reported by 31.43% (n = 11/35) of participants. Physical examination suggested possible alterations in the rotator cuff. Yet, no diagnosed cases were identified despite specific evaluation, in contrast to the high prevalence of rotator cuff injuries observed in the general population [17]. Most participants were young, which reduces the likelihood of degenerative changes typically associated with rotator cuff injuries. Additionally, physical examinations revealed that all maintained shoulder strength and their climbing activities, indicating that the shoulder pain experienced was not functionally limiting. Furthermore, no participants reported acute tear events or anterior luxation.

We found a significant relationship between dynamic climbing movements and shoulder apprehension, particularly in less experienced climbers, highlighting how evolving bouldering techniques contribute to new injury patterns. Although this association was not confirmed on the multiplicative scale, the strong effect observed on the additive scale and the high e-value suggest robustness against unmeasured confounders and its public health relevance. This finding aligns with recent trends in climbing injuries linked to more complex movement sequences, as noted by Eichler et al. (2023) [5].

Unlike overhead sports, where shoulder injuries are often associated with GIRD [6-8], our findings indicate that climbers, even those with shoulder subluxation, maintain balanced shoulder mobility (Table 8). This suggests that the unique biomechanical demands of climbing, characterized by dynamic and explosive movements, may drive a distinct injury pattern in the shoulder, differing from the mechanisms observed in other sports.

Elbow pain affected 11.4% (n = 4/35) of participants at the time of the study, with a cumulative prevalence of 14.3% (n = 5/35) diagnosed with lateral epicondylitis. These figures are consistent with prior studies, such as Josephsen et al. (2007) [15] (12%) and Müller et al. (2021) [2] (12.3%), underscoring the repetitive strain placed on the elbow joint during bouldering. Gripping and pulling movements inherent to the sport can exacerbate overuse injuries in the elbow, leading to conditions like tennis elbow.

This study has several methodological advantages, including combining self-reported data, detailed medical evaluations, and physical examinations. This dual approach enhances diagnostic accuracy compared to studies that rely solely on self-reports.

The results are most relevant to indoor bouldering populations in urban settings. Although we included climbers with various experience levels, caution should be exercised when generalizing these findings to other climbing modalities or outdoor environments, where different physical demands may affect injury patterns. Potential selection bias arises from the convenience and snowball sampling methods, which may limit the diversity of the sample.

Limitations in our study included the relatively small sample size, which might limit the ability to detect less common injuries and reduce the generalizability of the findings to a broader population of indoor boulderers. Additionally, the absence of MRI and dynamometric assessments means that our analysis relied on clinical evaluations and self-reports, which, while informative, may not capture every detail of musculoskeletal injuries. Lastly, by focusing solely on indoor bouldering, the applicability of our results to other forms of climbing or outdoor settings may be limited.

This study contributes valuable insights into the injury patterns of indoor bouldering and highlights the need for targeted prevention strategies. By capturing common and less severe injuries, this research provides a fuller picture of the risks associated with bouldering. Future longitudinal studies are needed to explore the long-term effects of bouldering on musculoskeletal health and to refine injury prevention approaches.

## Conclusions

The high prevalence of upper extremity injuries among indoor boulderers, particularly pulley injuries (22.9%, n = 8/35), anterior shoulder apprehension (17.2%, n = 6/35), and lateral epicondylitis (14.3%, n = 5/35), reflects the unique physical demands of the sport. Our findings suggest a significant association between dynamic climbing movements and increased injury risk, especially in less experienced climbers. These results highlight the need for targeted injury prevention strategies, focusing on strengthening and technique adaptation to mitigate the stress on joints and tendons inherent in bouldering.
